# Artificial Intelligence in Health Care—Understanding Patient Information Needs and Designing Comprehensible Transparency: Qualitative Study

**DOI:** 10.2196/46487

**Published:** 2023-06-19

**Authors:** Renee Robinson, Cara Liday, Sarah Lee, Ishan C Williams, Melanie Wright, Sungjoon An, Elaine Nguyen

**Affiliations:** 1 College of Pharmacy Idaho State University Anchorage, AK United States; 2 College of Pharmacy Idaho State University Pocatello, ID United States; 3 College of Pharmacy Idaho State University Meridian, ID United States; 4 School of Nursing University of Virginia Charlottesville, VA United States

**Keywords:** artificial intelligence, machine learning, diabetes, equipment safety, equipment design, health care

## Abstract

**Background:**

Artificial intelligence (AI) is a branch of computer science that uses advanced computational methods, such as machine learning (ML), to calculate and predict health outcomes and address patient and provider health needs. While these technologies show great promise for improving health care, especially in diabetes management, there are usability and safety concerns for both patients and providers about the use of AI/ML in health care management.

**Objective:**

We aimed to support and ensure safe use of AI/ML technologies in health care; thus, the team worked to better understand (1) patient information and training needs, (2) the factors that influence patients’ perceived value and trust in AI/ML health care applications, and (3) how best to support safe and appropriate use of AI/ML-enabled devices and applications among people living with diabetes.

**Methods:**

To understand general patient perspectives and information needs related to the use of AI/ML in health care, we conducted a series of focus groups (n=9) and interviews (n=3) with patients (n=41) and interviews with providers (n=6) in Alaska, Idaho, and Virginia. Grounded theory guided data gathering, synthesis, and analysis. Thematic content and constant comparison analysis were used to identify relevant themes and subthemes. Inductive approaches were used to link data to key concepts, including preferred patient-provider interactions and patient perceptions of trust, accuracy, value, assurances, and information transparency.

**Results:**

Key summary themes and recommendations focused on (1) patient preferences for AI/ML-enabled device and application information, (2) patient and provider AI/ML-related device and application training needs, (3) factors contributing to patient and provider trust in AI/ML-enabled devices and applications, and (4) AI/ML-related device and application functionality and safety considerations. A number of participants (patients and providers) made recommendations to improve device functionality to guide information and labeling mandates (eg, link to online video resources and provide access to 24/7 live in-person or virtual emergency support). Other patient recommendations included (1) providing access to practice devices, (2) providing connections to local supports and reputable community resources, and (3) simplifying the display and alert limits.

**Conclusions:**

Recommendations from both patients and providers could be used by federal oversight agencies to improve utilization of AI/ML monitoring of technology use in diabetes, improving device safety and efficacy.

## Introduction

Artificial intelligence (AI), a branch of computer science, attempts to build devices and software programs that explore and gather new knowledge, learn, and apply reasoning [1,2]. Machine learning (ML), a term often used interchangeably with AI, differs from AI in that in ML computer systems are able to adapt without following explicit instructions, using algorithms and statistical models to analyze and draw inferences from patterns in data [3,4]. Research in non–health care fields suggests that accountability is the most important attribute of AI, with fairness, security, privacy, and accuracy rated to have similarly high importance, and that transparent and comprehensible AI/ML systems are preferred [5-7]. Among the few studies that have explored patient perceptions of AI and related digital health applications, accuracy of decisions and patient empowerment have been identified as the 2 most important criteria [5,6]. In fact, a recent survey of health care workers in India found that technical skills, ethical concerns, and risk mitigation strategies were 3 key factors influencing perceptions regarding AI/ML use and that AI has a strong positive impact on patient cognitive engagement with health technologies [8].

As use of AI/ML in the health care arena is rapidly expanding, greater than expected benefits and patient outcomes have been seen [1]. Examples of AI/ML applications include but are not limited to diagnostic supports, image interpretation, tools that support rapid or automated data capture, and disease management [1,2]. In fact, recent studies have explored use of AI/ML in primary care [9] to support clinical decision-making and treatment management decisions for a number of chronic conditions, such as cardiovascular disease [10], mental health [11], and diabetes care [2]. However, little is known about how patients and providers feel about use of AI/ML in chronic disease management, if unmet AI/ML training needs influence AI/ML adoption, and most importantly, how barriers should be addressed (eg, labeling, training, and required supports). Left unaddressed, AI/ML concerns (eg, potential interpretation errors and data privacy issues) and use in nonrepresentative samples (eg, educated, well-resourced populations), could contribute to lack of patient and provider trust in AI/ML applications, health inequities, reduced efficacy, and poor patient outcomes, as well as preventable safety concerns [7,12,13].

The US Food and Drug Administration (FDA) is responsible for protecting public health by ensuring the safety, effectiveness, quality, and security of drugs, biological products, medical devices, and software (eg, mobile health apps) [14]. In 2014, the FDA established the Patient Engagement Advisory Committee (PEAC) to ensure safe and effective AI/ML implementation in the health care setting. The PEAC, made up of patients and providers, is responsible for premarket review of AI/ML devices, guiding device labeling requirements, and supporting “transparency and real-world performance monitoring” to ensure safe and effective AI/ML use from premarket development through the postmarketing period [14,15]. The primary objective of this qualitative inquiry is to build upon the work of the FDA and PEAC to (1) understand general patient AI/ML information needs, (2) understand factors that influence patients’ perceived valuing of and trust in AI/ML devices to support diabetes management, and (3) guide current and future FDA AI/ML labeling requirements to ensure the appropriate information is accessible and supports safe and effective use of AI/ML-enabled devices.

## Methods

### Overview

Barriers to technology utilization (eg, understanding, access, and perceived need) differ by population and geographic region (eg, access in rural, underresourced, and ethnically diverse communities) [16,17]. Patients (and providers) may have limited awareness of the many AI/ML applications available to support patient health management. Assumed AI/ML application complexity, novelty, and costs make it difficult for patients to recognize and communicate their reservations and management needs with providers (eg, their general perceptions of the value of the relevant technologies, unmet information needs, necessary regulatory concerns, and assurances required to trust AI/ML applications) [17-20]. Due to the variety and relative maturity of available AI/ML diabetes management and prevention applications, we chose to focus on perceptions, information, and implementation needs of patients and providers considering and using AI/ML applications to manage their diabetes.

### Setting

To understand general patient perspectives and information needs related to the use of AI/ML in health care, we conducted a series of 9 focus groups and 3 interviews that included a total of 41 patients and interviews with 6 providers, including nurse case managers, pharmacists, physicians, and an endocrinologist serving 3 different patient populations in Alaska (n=9), Idaho (n=23), and Virginia (n=8). Within the context of this study, members of the research team and target research population were part of the community of interest (individuals with type 1 or type 2 diabetes, their caregivers, and health care providers managing diabetes) and familiar with the needs of the patients with diabetes. Project team members have conducted similar qualitative studies in the past and understand the health care access and resource disparity barriers (eg, education, transportation, and financial deficits) that exist for patients and providers living in underresourced, underrepresented rural and urban communities across Alaska, Idaho, and Virginia.

### Approach

To ensure consistency in the data collection process, a moderator’s guide was developed to facilitate and standardize the focus groups and interviews. Guided by the established health technology assessment literature, the moderator’s guide scenarios and questions were developed and drafted by the research team and focused on (1) participant understanding of smart products and devices that use AI to manage diabetes, (2) information needs to effectively and safely use AI/ML applications, and (3) participant suggestions on how best to communicate the necessary information to patients and providers to safely and effectively use applications and devices. For each application or device, we generated a patient-friendly description of the technology and how AI/ML was used. We generated context-specific queries for each example. Questions assessed patient and provider information needs, expected regulatory or other assurances, trust, and general perceptions of the value of the application. Scenarios were tested and refined during pilot sessions with a set of 4 patients and a provider. Questions were posed to providers in semistructured interviews that were similar to those asked of patients; the questions focused on information needed by patients to safely and effectively use AI/ML applications for diabetes management.

All focus group sessions and semistructured interviews were conducted by trained team personnel (RR, CL, and IW) who understand diabetes management challenges patients and providers face, know how to think about the problem (ie, reflexivity), and are sensitive as to how the data collection process may shape individual- and community-level responses (ie, research problem framing). This unique combination of professional experience, health training, and community engagement supported a more comprehensive understanding of training needs, sustainable training program development and implementation, and took into account prior assumptions, factors (ie, social contextual inquiry), and approaches used by the team (eg, diabetes and device information sharing) to overcome limited patient and provider AI/ML understanding and identify and recognize unmet information needs due to limited device and system experience [21].

RR, the qualitative research lead, conducted a 60-minute Zoom-based training session with all research team members to ensure focus group and interview consistency. Pilot training sessions were recorded, providing pertinent technology-based examples that focused on unmet patient and provider training needs (ie, use, maintenance, and troubleshooting), device safety concerns (alerts, warnings, and functionality), preferences for device testing, information sharing concerns, and other factors directly and indirectly related to device use (trust).

### Ethical Approval

This study was granted expedited approval with a waiver of written consent (IRB-FY2021-259 for the work with patients and IRB-FY2021-260 for the work with providers) by the Idaho State University Institutional Review Board (IRB) and is subject to university research governance procedures. The Idaho State University IRB was also approved as the single IRB of record for the University of Virginia site. Participants or their legal guardians verbally consented to participation at the time of the interviews or focus group scheduling. Verbal consent was confirmed and documented again prior to the interviews or focus group initiation. All research was performed in accordance with relevant guidelines and regulations applicable to human subject participation and the Declaration of Helsinki.

### Theoretical Framework

The Consolidated Framework for Implementation Research (CFIR) provides a menu of distinct constructs associated with effective program implementation (eg, implementation and organizational climate, culture, and context) and systematic analysis, and it supports incorporation of organization findings into practice [22,23]. Implementation climate, our primary construct, focuses on the impact that climate has on the implementation of innovative and progressive services, and the extent to which organization members perceive that an innovation is expected, supported, and rewarded by their organization or community [23-26].

### Participant Selection

To recruit patients with type 1 or type 2 diabetes, flyers were distributed through local community groups, health care clinics, and diabetes educators. These groups included, but were not limited to, the Diabetes Alliance of Idaho, Camp Hodia, Idaho Primary Care Association, Community Council of Idaho, local community venues (churches and libraries), and local health care clinics (St. Luke’s Endocrinology, Idaho Nutrition Associates, Idaho State University clinics, Full Circle Health, and University of Virginia [UVA] Health). The flyer was also shared with Facebook groups, including the Juvenile Diabetes Research Foundation Idaho, Native American Coalition of Boise, and Latter-Day Saints church groups. Lastly, the flyer was also promoted through paid promotion on Facebook. Paid promotion targeted the southern Idaho and Anchorage, Alaska, areas.

The flyer contained information regarding the study purpose, focus group eligibility, compensation, investigator contact information, and a screening survey link for interested individuals. The screening survey included full study details and collected eligibility and contact information. After individuals completed the screening survey, the project coordinator or research team member called them to confirm their interest in participation, reviewed consent, collected necessary information (ie, participant age, gender, diabetes diagnosis, race/ethnicity, technology use, and education level) and enrolled them. Participants could also complete the consent paperwork electronically or use paper forms, in person, before the focus group or interview. We used inclusive focus group methods to ensure participants’ psychological safety and to encourage engagement. Two sessions had a majority of African American participants and 1 session had a majority of Native American/Alaska Native individuals.

Investigators used their relationship with area providers to recruit participants. In addition to these established relationships, area providers were also identified through an online search and contacted via email. We sought to recruit both physicians and certified diabetes care and education care specialists (CDCES) who care for patients with diabetes. After providers expressed their interest and willingness to participate in interviews, screening paperwork was completed, and their consent was verbally obtained prior to beginning the interview. We conducted semistructured interviews with providers using the established moderator’s guide and Zoom, an online meeting platform. All focus group and interview sessions were audio-recorded and transcribed. Individuals received a US $75 gift card as an incentive for their participation.

### Data Analysis

Grounded theory guided data gathering, synthesis, and analysis [27-29]. Thematic content and constant comparison analysis were used to identify relevant themes and allow for general and across-group assessments for both exploratory and verification purposes. An inductive approach was used to link data to key concepts, including patient perceptions of trust, value, accuracy, transparency, assurances, and preferred patient-provider approaches to application interaction [28,29]. QDA Miner (Provalis) [30] qualitative coding software was used for analysis. During the first stage of analysis, each transcript was systematically coded by at least two coders, with an initial codebook created based on moderator questions and initial review of the transcripts. Data were chunked into smaller units, definitions were established for each code, and the code/definition was attached to each unit (open coding). During the second stage, codes were grouped into categories (axial coding). Lastly, in the third stage, the researchers met frequently to refine and finalize codes (selective coding), identify discrepancies, achieve consensus, and establish the final codebook. Two coders systematically coded the data generating descriptive and analytic themes and identified patterns and dominant concepts that emerged during analysis. Where possible, codes associated with responders (ie, patient characteristics) were also included (Multimedia Appendix 1).

Representative quotes were sorted by codes, summary descriptions for each code were written, and information was linked to demographic data to identify additional patterns and themes. Preferred information or labeling presentation approaches and desired content were categorized and cross-referenced to patient classifications and themes were identified and prioritized. We used progressive analysis (data analysis concurrently with data collection) to support selection of scenarios and decisions on when enough sessions had been completed to achieve saturation in qualitative responses to key concepts [27,28,31]. Our full team of investigators reviewed (and iterated as needed) definitions, coding rules, and emerging themes (within the context of relevant interviewee quotes) for rigor, credibility, authenticity, sensitivity, and thoroughness [31]. The Consolidated Criteria for Reporting Qualitative Research (COREQ) were used to ensure comprehensive reporting of the qualitative data [32].

## Results

### General Characteristics

Between August and October 2022, we recruited and interviewed 41 patient participants (Table 1) to participate in 1 of 9 patient focus group sessions, 3 patient interview sessions (it should be noted that with teenagers, we conducted one-on-one sessions due to after-school conflicts), or 6 provider interviews. Provider interviews consisted of 3 pharmacists or CDCES, 2 primary care providers, and 1 diabetologist.

**Table 1:** Participant demographics (N=41).

Characteristics			Values
**Age category, n (%)**			
	Adults (aged 20-89 years)	38 (93)	
	Teenagers (aged 16-19 years)	3 (7)	
Age, (years), mean (SD)			48.4 (20.4)
Age, (years), median (IQR)			48 (32-66)
**Gender^a^, n (%)**			
	Male	19 (46)	
	Female	21 (51)	
**Diabetes type, n (%)**			
	Type 1	17 (41)	
	Type 2	24 (59)	
**Race^b^, n (%)**			
	Alaska Native/American Indian	7 (17)	
	Black	13 (32)	
	White	24 (59)	
**Advanced technology user^a^, n (%)**			
	Yes	33 (80)	
	No	7 (17)	
**Education level, n (%)**			
	Some high school	3 (7)	
	High school, General Educational Development test, or equivalent	6 (15)	
	Trade school, apprenticeship, or equivalent	4 (10)	
	Associate’s degree	8 (20)	
	Bachelor’s degree	9 (22)	
	Postgraduate or professional degree	11 (27)	

^a^Data for 40 participants only; percentages are of 41 participants and do not add up to 100.

^b^Not mutually exclusive groups; percentages do not add up to 100.

### Themes, Subthemes, and Representative Quotes

Representative quotes are provided with relevant codes, themes, and subthemes: information needs (Multimedia Appendix 2), safety (Multimedia Appendix 3), and trust (Multimedia Appendix 4). Information needs were broken down into general needs, as well as training and informational support needs, preferences for information sharing, sources of information, troubleshooting, and information maintenance needs. Themes, subthemes, and representative quotes highlighted in Multimedia Appendix 2 emphasized the importance of patient training and ready access to necessary information tools and resources, especially in response to AI/ML application alerts and warnings. Participants requested that information and training be provided in a number of different ways (eg, pamphlets, in-person training, computer-guided supports, and sharing of patient experiences). Multimedia Appendix 3 presents safety concerns and needs identified by participants. Suggestions focused on input controls, alerts, reporting, override functions and manufacturer labeling, information, and device mandates that could increase safety and improve AI/ML application trust. Lastly, Multimedia Appendix 4 shows factors affecting participant trust and use of AI/ML applications. Reliability and accuracy of the measures in the specific population, AI/ML application limitations, and the impact of endorsements on trust are presented.

## Discussion

### Principal Findings

In health care, use of advanced computational methods and related AI/ML applications is expanding [1,2]. Provider- and patient-facing devices and applications (eg, continuous glucose monitors, insulin pumps, electronic health record–integrated decision supports, and mobile health apps) show great promise for improving diagnosis, data interpretation, and use of data to support treatment recommendations, dosage adjustment and management, and risk assessment [33].

While there is emerging research on public perceptions of responsible AI/ML application use, in general, little is known about how user interaction with specific AI/ML applications or related system information (eg, labels, intended use statements, and warnings) influences patient and provider perceptions of performance and addresses the ethical concerns or risks related to AI/ML use, especially in diabetes management and tailored medication therapy [2,6,34,35]. In order to provide useful guidance related to the representation of AI or AI-related explanations to patients with diabetes, it is important to explore patient and provider understanding of AI/ML applications, identify safety concerns with AI/ML use, and address underlying mistrust of AI/ML devices to support realistic contexts of use. In our research, we identified themes and subthemes and present summary descriptions, representative quotes, and relevant respondent data that identify and highlight the diverse patient and provider perspectives on unmet or suboptimal AI/ML application information and training needs, unaddressed safety concerns, and factors that influence patient and provider trust in the use of AI/ML applications for diabetes management.

### Information and Training Needs

As we are all aware, diabetes is highly prevalent in the United States, affecting approximately 10% of Americans and 27% of people aged over 65 years [32]. The potential for AI/ML applications to improve outcomes for people living with diabetes is significant; however, information and training are necessary to support the human factors associated with safe and effective AI/ML application use in diabetes management, especially in older adults [35-37]. Patients need to understand all metrics displayed on the device to safely and effectively manage their diabetes. In our qualitative work, we found many patients rely on health care professionals as their primary resource for information about the appropriateness, quality, and safety of selected diabetes management technology. Most health care professionals may not have the necessary knowledge and experience with all available technology platforms to support meaningful use and troubleshooting of AI/ML applications for diabetes management; therefore, they require external support. In fact, according to a technology review conducted by the United Kingdom’s National Health Service, rapid technological change requires that all health care providers (eg, doctors, nurses, pharmacists, and paramedics) receive extensive technology training [38].

This finding is consistent with the literature exploring patients’ and health care professionals’ perspectives toward technology use in diabetes management [39] and the concerns regarding safe and effective use of available technology that may be exacerbated if and when AI/ML applications become more available to patients (ie, over-the-counter and prescription applications) [40]. Therefore, it is essential that both patient and provider information and training needs are addressed to ensure patient diabetes management and safety needs are met by AI/ML device use (eg, understanding of device functionality, data availability, and safety functions). In fact, most participants in our study wanted and needed more information about the device or application than they initially received during training (eg, what it was measuring, why it was measuring it, and how results would be used to improve their health). Patients requested that device information be clear, concise, and written in lay terms and that comprehensive information be provided in a number of different ways (eg, in-person training, hands-on device training, real-world instructional videos, manufacturer videos clips and targeted frequently asked questions, pamphlets, and cheat-sheets) to accommodate different learners and learning styles. Many patients also requested that peer-to-peer training and evidence-based informational resources be provided to support real-life device use and troubleshooting. We also found that the amount of information provided at any one time was often a limiting factor and was both overwhelming and confusing to the patients and caregivers. It is important to note that initially, patients in our study were unsure of their own information needs, and that questions arose with daily device and application use over the following weeks. This suggests that a tiered or layered approach to teaching [41], validated and used in adult learning and education models, be included. Maintenance, troubleshooting, and potentially life-threatening alerts might be necessary to ensure appropriate and safe device use. A number of patients and providers in our study suggested a tiered approach to both knowledge assessment and functionality, which would require a minimal level of disease state and device or application knowledge to allow users to enable specific functions. The staged or tiered approach to training was viewed by many patients as an effective and efficient training mechanism aligned with patient understanding. The ability to watch instructions in segments was thought to allow for device mastery. Patients also requested the ability to trial a number of devices and to be connected to all relevant systems to ensure that the device is appropriate for them (eg, considering type of diabetes and experience with technology). This is consistent with patient training needs and requests seen in literature regarding human factors and usability engineering for medical device labeling and function, especially among older adults [36,39,42].

Lastly, there were a number of participant suggestions regarding training and support that could be provided by device manufacturers to improve device use and testing. Suggestions included the following: (1) provide a basic starter guide for the first few days of use; (2) provide practice devices that allow for hands-on trials; (3) provide links to online resources, local supports, and reputable community resources (eg, professional organizations, blogs, and personal reviews) on the manufacturer website; (4) provide 24/7 live in-person or virtual emergency support; and (5) provide brief, searchable, instructional resources, such as videos indexed by problem and answers to frequently asked questions.

### Safety

In respect to safety, patients in our study were most concerned with (1) having a clear understanding of alerts and warnings, (2) being able to recognize and rapidly respond to a potentially life-threatening situation (eg, device overrides, function lockdowns, and system-down alerts), (3) knowing immediately if there were device connectivity issues that impede overall diabetes management (eg, the continuous glucose monitor not connected to the insulin pump), and (4) having safeguards to reduce the risk of user error (eg, data field restrictions and order entry confirmation requirements).

Participants wanted access to real-time, live device safety support offering them the ability to more effectively and efficiently troubleshoot issues with devices that directly control insulin delivery. Participants also voiced concerns regarding the number of alerts they received, the alert descriptions being provided as codes, the information provided by the manufacturer or provider about what to do to address the alert (device instructions), and mechanisms in place to stop alerts once the patients has addressed them (to avoid alert fatigue). This is consistent with the scientific and lay literature; having clear predictive and real-time alerts is important but so is ensuring that alerts can be tailored to patient needs and address provider concerns [43-45].

Providers stressed the importance of patients having access to a limited number of clear, clinically important alerts and necessary alarms and the provision of patient education focused on understanding what to do in the case of an alert or alarm. If users cannot see or interpret the alert, they will not respond appropriately, a documented challenge for many older adults [37,46]. In order for required safety information provided to patients to be useful, it needs to be immediate, detailed, and prescriptive and provide simple instructions to the patient and caregiver [47,48]. It is also important that device updates related to safety and device functionality be pushed out automatically to ensure continued safe and effective device and application use. Lastly, it was recommended by participants that all safety features need to either remind or directly connect patients to providers, emergency services (eg, 911 and Medic Alert), and necessary troubleshooting resources to help support patient understanding and encourage patient ownership of care.

### Trust

Trust in the device or application was based on trust in the health care provider’s recommendations and the participant’s experience with that health care provider; however, it also extended beyond the clinical interface to the collection, collation, and use of personal data [49-53]. In our study, individuals consistently treated by the same health care provider or specialist appeared to have more trust in the provider-recommended device. However, it is important to note that concerns regarding blind trust were voiced by a number of patients and providers in our study and that trust in the device was directly related to patient experience, device accuracy, and duration of device use.

AI/ML application use can be associated with a number of risks as well as benefits. As such, our findings are supported by other research that emphasizes the complexity of and need for trust being embedded in all aspects of AI. Specifically, Lockey et al [50] support this finding, showing that transparency, explainability, and accuracy metrics are important, although they may not be sufficient, to garner trust in AI applications. In line with our methodological approach, Lockey and colleagues [50] also suggest the need to examine multiple key stakeholders in relation to AI systems and their varying expectations and alignment with the outcomes of using the AI device.

Participants expressed the need for exposure to the device and a mechanism in place to double-check readings and functionality to build trust; they also expressed to need for the opportunity to question device results and troubleshoot concerns with providers and other health care team members. Participants raised an important point on having detailed and accessible information on the population characteristics (ie, age, race/ethnicity, gender, and diabetes type) of those who tested the device or application. Participants wanted to know that the device was tested in individuals similar to them. These results are in line with best practices for ensuring and promoting trust in AI implementation, such as including representative and equitable populations in its development, having a user-centered design, and ensuring constant accountability of the algorithm being used to maintain accuracy [51]. Given the importance of human factors and the associated patient outcomes in use of AI devices, it is essential to understand how trust is linked to the needs of the user and design requirements [52,53]. Our data support optimizing the opinions of patients and users and acknowledging that trust shapes clinicians’ and patients’ use and initial adoption of AI devices [52].

The implementation of the strategies discussed above can increase proper use, safety, and trust regarding AI-enabled medical devices. In an informal review of patient-facing AI systems available from the FDA [54], we found that current apps and systems lack detailed information and resources for users, both patients and providers. We believe this makes our findings even more important. As manufacturers and device makers hopefully integrate our suggestions, real-world examples will arise. Further investigation will then be needed to optimize AI system interfaces.

### Conclusions and Next Steps

Our work supplements the emerging literature related to public perceptions of responsibility and ethics in AI/ML device and application use [7,13,14]. We hope that our findings inform the FDA’s decisions on public health and safety related to AI/ML devices and applications. AI/ML applications demonstrate a great deal of promise; however, even greater outcomes will be realized if ethical and responsible AI design engenders greater engagement and use by all. It is important to understand how to present information to patients about AI/ML characteristics identified as important to them, such as data privacy, fairness, accuracy, and risks.

## Appendix

Multimedia Appendix 1 Codebook.

Multimedia Appendix 2 Themes, subthemes, and representative quotes related to information needs.

Multimedia Appendix 3 Themes, subthemes, and representative quotes related to safety.

Multimedia Appendix 4 Themes, subthemes, and representative quotes related to trust.

## Abbreviations

AI: artificical intelligence
CDCES: certified diabetes care and education specialist
CFIR: Consolidated Framework for Implementation Research
COREQ: Consolidated Criteria for Reporting Qualitative Research
FDA: Food and Drug Administration
IRB: institutional review board
ML: machine learning
PEAC: Patient Engagement Advisory Committee
UVA: University of Virginia
