# Supplementary material for: Artificial Intelligence in Health Care—Understanding Patient Information Needs and Designing Comprehensible Transparency: Qualitative Study
Source: JMIR AI. 2023 Jun 19;2:e46487. doi: 10.2196/46487 (PMC10851077; doi:10.2196/46487)
Supplement: Multimedia Appendix 1 [file ai_v2i1e46487_app1.docx]

### Multimedia Appendix 1. Codebook

|  | Code | Definition/Description |
| --- | --- | --- |
|  |  |  |
| **Information** |  |  |
|  | General Information | Patient information needs requirements to use technology and understand results (e.g., timely, accurate, accessible, clear, concise, searchable) |
|  | Preferences for Information | Patients and or providers preference to get information about device and /or application |
|  | Sources of Information | Where individuals (patients and or providers) prefer to get information about device and /or application |
|  | Sharing of Information | Who should information collected from the device and/or application be shared with |
|  | Training and Informational Support Needs | Supports patients are requesting/needing for safe and effective device use |
|  | Troubleshooting and Maintenance Information | Required device maintenance and troubleshooting training and information supports (needs and preferences) |
| **Device** |  |  |
|  | Device Preferences | Patient preferences for the device and/or application |
|  | Device Alerts and Warnings | Preferences for alerts provided by the device to the patient (Note: alerts related to effect, safety, etc.) |
|  | Device Choice - Options | Having control over the healthcare decision and/or device use options (e.g., ability to tailor device and/or application). |
|  | Device Algorithms and Functionality | How the device or application works (e.g., how calculates dose, how it is calibrated, etc.) |
|  | Device Compatibility and Connectivity | Device compatibility and connectivity (e.g., communication between devices and related concerns) |
|  | Device Creation and Testing and Population | Information regarding how the device and/or application was created and tested, including study population characteristics |
|  | Device Information Feedback | Concerns related to information feedback not related to connectivity |
|  | Device Access | Barriers to device and / or application access |
| **Safety** |  |  |
|  | Safety Needs | Information and features needed by patient to feel safe using the device, includes alerts and features to reduce errors |
|  | Safety Preferences | Preferences on how to share information about safety |
| **Trust** |  |  |
|  | Trust Accuracy | What is necessary to trust the device or technology function and/or results |
|  | Trust Endorsed By | Who is endorsing the device and/or application, required to trust |
| **Challenge** |  |  |
|  | Biggest Challenge | Biggest challenge to device and/or application utilization identified by patient or provider |
