# Supplementary material for: Artificial Intelligence in Health Care—Understanding Patient Information Needs and Designing Comprehensible Transparency: Qualitative Study
Source: JMIR AI. 2023 Jun 19;2:e46487. doi: 10.2196/46487 (PMC10851077; doi:10.2196/46487)
Supplement: Multimedia Appendix 2 [file ai_v2i1e46487_app2.docx]

**Multimedia Appendix 2.** **Information Needs**

|  | Themes | Sub-Themes | Representative Quotes |
| --- | --- | --- | --- |
|  |  |  |  |
| **General Information** |  |  |  |
|  | Training needs | Importance of training | 8/10, quite a bit more training [then device that provides recommendation]. |
|  | Information needs | Requirements | I want to know, is there any action that's required on my part? |
|  | Information needs | Predictions | I am always on the go so trying to plan ahead is really important to me. It is helpful to know that this might happen in this much time ...I like having predictions. |
| **Preferences for Information** |  |  |  |
|  | Training preferences | Amount of information provided | I just want to know how does it know what to give to me, if [my blood sugars] are going up or down, ...and connections between [devices]. |
|  | Training preferences | Information quantity | I think some people like to know every detail and some don't, so it is really figuring out your level of interest [information needs]. |
|  | Information preferences | Information quantity | Individual importance seems quite variable, some trust that since it’s on the market, it must be trustworthy, others seem to treat it like a PubMed article or something, wanting to know literally everything about it. |
|  | Information preferences | Information Accessibility | I think it would probably better to have some kind of pamphlet that's out, because a pamphlet, usually it's got a picture or something, or the words are bigger, because the little ones that they insert in your device labels are so small, you're just like, "Well, I ain't got time to read that. Ain't got time to find my glasses and read that." You don't pay attention to it. But if you got a pamphlet, that's got a nice picture on it, it's got some bold color too, you [will] … read it. |
|  | Information preferences | Information Accessibility | Yeah, I think that [information about how the application calculates dose] should be included in the history, when you're putting together a video, all that information, the calculations, and so on. |
|  | Training preferences | Information Accessibility | Oh my gosh. I would say probably a tutorial kind of thing, especially for the younger generation, since they’re technology savvy. For the older generation, I think that you would have to have the nurses to show them how to do this because some of them don't even understand technology or how to use a phone. |
|  | Information preferences | Guidance and Interactions | I feel like there should be an option that says contact your doctor or whatever, if it gets to a point where it’s just, the person just feels a little bit off, they're not agreeing with what's being advised and everything. There should be an option for them or some pop-up or something that says contact your local physician or something like that. Just so that it gives them an out because sometimes people will rely on too much of the information or the app that they forget that, "Oh yeah, I can pick up the phone and call." |
|  | Information preferences | Alerts and Warnings | Ability to turn [alerts and warnings] on and off. Because sometimes you're on schedule and sometimes you're not, it just depends on how busy you are. I mean, everybody's life is different and we all have our own different things like me I am so busy. I get caught up in schoolwork, being a mom and then I'm tutoring sometimes. And I was like, "Did I do this?" And then by the time I'm feeling all woozy, I'm like, "Yeah, my sugar is low. So, let me do something about this before I'll be sitting on the couch." |
|  | Information preferences | Guidance and Interactions | I like the interactive as well you know, but I don't know that I would want to go somewhere to do the interaction I think I'd be happy being on you know, a 15 minute one on one. With a case manager or whatever product control specialist whatever to help me set it up, make sure it's running. Make sure I'm comfortable with it and then maybe follow up with me and see if I'm doing all right with it. |
|  | Information preferences | Guidance and Interactions | A lot of times they're like, okay, describe exactly what's on the screen, and you can do that, and it matches up and um, I also think what would be kind of cool, if it was possible, is, do like a zoom thing, if necessary, where you could, you know, meet with them and be able to like literally hold up your pump, or whatever. If it's having a problem, you'd be like, “This is what's happening. This is the alert that's coming. What does this mean?” |
|  | Training preferences | Tools and Resources | A training [about alerts] for sure, would be beneficial, and then, honestly, like a little cheat card [with all the alerts and their meaning], would be kind of nice. If it was just like a little index card or something like that. You know that someone could easily put on a fridge, or even just to have on their bedside table or something when they're first using it, so that if some of those alerts pop up and they're like, “Oh, yeah, I don't remember what that one means.” |
|  | Information preferences | Tools and Resources | Being able to go on to the manufacturer's website, and it being readily and easily available for maybe someone not so tech savvy, such as in the frequently asked questions on the top of the website. That works awesome. I don't know just, being able to really easily find it, not having to dig or use a search function, just being able to go to the manufacturer's website and know that I trust them enough to be able to find it easily, or even being able to call the non-emergency help desk, and then being like “Oh, this is where you find it.” |
|  | Training preferences | Tools and Resources | [I want access to training by] videos. And then I think you mentioned something about crowdsourcing. I'm on a bunch of Facebook groups where having all those people that use that device going on there and saying, "Hey, this weird thing happened to me. Has it happened to anyone else?" And then having them share their experiences is completely invaluable because sometimes the support reps that you get or the trainers, they either don't wear it or they don't have diabetes themselves. They might have someone in their family that does, but they don't have that same experience as everyone else. |
|  | Information Preferences | Alerts and Warnings | Sometimes there's too many alerts, too, I mean on my insulin pump. It gives me an alert anytime my number is just like, if it reaches two hundred, I get three alarms, and I don't need three alarms. I need one, and I can't change it, because it gives me one through my continuous glucose monitor, one through my pump, and then one through my control. |
|  | Information Preferences | Device Standardization | You know there has to be some standardization of the commands and the buttons and the layout, the format, so that, regardless the provider can come in and actually say, “Oh, yeah, when you had your seizure an hour ago your blood sugar was twelve”, Mm-hmm but I can't, I’m not competent enough because I don't know that specific model that specific machine to just pick the thing up and start messing with it and get that information back. A lot of times I got that patient we go, “Can you pull up your records for the past twenty- four hours?” It'd be very helpful to think it's standardized. |
| **Sources of Information** |  |  |  |
|  | Information Source | Providers | Just for my doctors. |
|  | Information Source | Professional organizations | When considering different devices, the best way to share information is a diabetes website |
|  | Information Source | Community Resources | Most of it has been googling and finding forums, and what people have said and talked about their experience. Obviously, that's going to be a different level of credibility than manufacturer or my doctor, but if other people have had a similar experience, I think it has some value. |
|  | Information Source | Information Accessibility | I think it would probably better to have some kind of pamphlet that's out, because a pamphlet, usually, it's got a picture or something, or the words are bigger, because the little ones that they insert in your device labels are so small. |
|  | Information Source | Personal Experience | A lot of my experience is just based on my own devices that I wear, but anytime I have a problem with my device or something I can call. I also know I can email them [ the manufacturer]. And I also know that there’s a website where I can go through various problems. |
|  | Information Source | In-person | [A place] where we could come in and we could attend and we could ask questions and see demonstrations. |
|  | Information Source | Interactive Experience | I like the interactive as well you know, but I don't know that I would want to go somewhere to do the interaction. I think I'd be happy being on you know, a 15 minute one on one with a case manager or whatever product control specialist whatever to help me set it up, make sure it's running. Make sure I'm comfortable with it and then maybe follow up with me and see if I'm doing all right with it. |
|  | Information Source | Provider | I want to get it [information] directly from my doctor. |
|  | Training Information | Community Resources | I feel like give me all the information, but until I start using it and playing with it, then go back and have some questions, and maybe call the rep that showed me how to use it. Or my doctor, or just getting online and doing the frequently asked questions or blogs. |
|  | Training Information | In-person | I personally like the [device] training with the person first, so I can have an opportunity ask questions, and then however else you want to give it to me for support. |
|  | Information Source | Computer-guided recommendations and interventions | The little word paper clip, like make recommendations on how to write your paper, [could be used to give you information about] your pump. |
|  | Information Source | Community Resources | I feel like there needs to be a database for the past experiences of people that have used the app and how it worked for them. I think if there's a database where people with diabetes can relate so as to relate their experiences with the app, so that moving forward, we'll be able to navigate it properly. |
|  | Information Source | Access and Availability | I know there's availability, but I haven't done it, is like a live chat on certain devices, they go to the website and there's a live chat with somebody or video chat with somebody. |
|  | Information Source | Computer-guided recommendations and interventions | Nice if it [information on maintenance and troubleshooting] was actually a link on the phone on the APP of like “I'm having a problem press this button” and it calls directly the manufacturer of that product or it takes them to the websites. They do a video or even walking through like a decision tree of like what's going on.  Okay well here's the video to help you solve this problem. |
|  | Information Source | Computer-guided recommendations and interventions | I mean, if they're [the patients] up to accessing a website, a website with frequently asked questions, or a place to, you know, type in questions that they have. |
|  | Information Source | Access and Availability | I like when the manufacturer has like a 24-hour seven day a week hotline that someone answers the phone and walks them through it. They're really like the experts. |
| **Sharing of Information** |  |  |  |
|  | Information sharing | Manufacturer | Yeah, let the manufacturer have the information so they could make the technology better. The more they know the better |
|  | Information sharing | Family | I care who has [my data]. Like my mom and dad and doctor have my information. That's all the people I want to have [information] I don't want anyone else and some random person. |
|  | Information sharing | Healthcare provider | I care who has [my data]. Like my mom and dad and doctor have my information. That's all the people I want to have [information]. I don't want anyone else and some random person. |
|  | Information Sharing | Manufacturer-use | [I am ok sharing formation with the manufacturer as] long as it doesn't infringe on my safety and security. I mean, just information, that's fine. They don't have to know who it came from. |
|  | Information Sharing | Use-guided Sharing | So, the data you got now can help somebody else in the future [this is ok to help refine algorithm or device]. |
|  | Information Sharing | Insurance and Payers | I feel like when it comes to insurance, that they should not be able to just freely have that information. I think that it's something that I would need to be able to give them the information. |
|  | Information Sharing |  | Nobody, no one [I don't have a problem with sharing my information]. |
|  | Information Sharing | Manufacturer-use | I'd let them [device manufacturers] have the information. Yeah, so they can make better technology. |
|  | Information Sharing | Consent and Permissions | I'm generally okay with it [sharing information]as long as I know, and I'm able to in some way like, give consent. I mean, if it's something that they're just taking the numbers, and it has nothing like, there’s no connection with me. It's like, okay, and they're just numbers ultimately like they're not going to have any silly connection to me. But if it's like, they're taking a little bit more about like my profile, whether it be, you know, this is a female. She's nineteen years old, or whatever it may not have my name. But if they're taking a little bit more detailed information, I like to be informed about it, and be able to give consent, if possible. |
|  | Information Sharing | Consent and Permissions | For one thing, they [the manufacture] need to ask your permission, so you agree or disagree with that, but I feel like they are using it better. All of us that have type one diabetes, because they can, with some of the stuff that happens with us, they can get more knowledge to make better devices. …so use if they ask permission, then you are comfortable with them, using your information. |
|  | Information Sharing | Insurance and Payers | If they're [insurance company] using the information to help others right, to help the machine learning or to help the artificial intelligence learn right and adapt and change to help others kind of, the rest of you feel about that I would love nothing more than to have my information used for the betterment of treatment. |
|  | Information Sharing | Consent and Permissions | I care on two points [what information the device collects from me that it is], de-identified and is it [information] sold. |
|  | Information Sharing | Consent and Permissions | I don't care if it's [my information] used for research purposes, academic research purposes, whatever. But if it is sold to a third party, I want to know that that may happen, and then I want it de-identified. |
|  | Information Sharing | Providers | I feel like you should stop at your healthcare provider or someone that you have a relationship with and no one else needs to know anything about it. If they request information to improve their product and you consent to that, sure, if that's your choice, but not just suck data from everyone that uses the device |
|  | Information Sharing | Community-Guided | That was really frustrating. I wanted somebody to ask me, and I never got asked. I didn't tell anybody. So sometimes I want to share that information so that it'll help others and help the improved pumps in the future or whatever the technology is. |
|  | Information Sharing | Consent and Permissions | As long as there's nothing identifiable back to me specifically, then [sharing data is okay], because I think it's actually a good thing if people will provide that data so they can improve things because they can see what works, what doesn't work |
|  | Information Sharing | Consent and Permissions | I first want to know with who it's [the patient’s data is] being shared. Is it just, you know, is it just the company? Because again, you're using their portal. Is it being shared with third parties? Is it being shared with the insurance companies? Are they looking at their compliance? So, I think those would be important things to know. Has it been shared with payers? |
| **Training and Informational Support Needs** |  |  |  |
|  | Information needs | Timing/length training | [Timing of information provision based on device access] I feel like give me all the information, but until I start using it and playing with it [I won't fully understand], then [come] back when I have some questions, and maybe call the rep that showed me how to use it. |
|  | Information needs | Timing/length training | Segmented or tier training I didn't know how to use [my pump] for a while, because we didn't have time to cover everything in that first session, [I would prefer] something I can reference specifically piece by piece rather than this big long “here's everything [training].” Let's sit and go through all of it at once...I lose interest. |
|  | Information needs | Initial training needs | I like probably a basic starter guide, of “here's how to do it for a couple of days” and then something that's not too overwhelming, but then maybe something I could click through online on the website, videos or something interactive that goes more in depth on some of the advanced features. |
|  | Information needs | Type of training | I do much better with an in person [training], show me how to use device. |
|  | Information needs | Type of training | Consensus for training media to be digital, also wants available for download for when wifi sucks or something else [is going wrong] |
|  | Information needs | Options | I think having both options available because some people will look into it [how the app was created] and take the time to read it. Some people want to actually have the ability to download it and print it and actually read about it. I think having a variety of options available because every person learns differently too. |
|  | Information Needs | Options | It's nice to have a video, but it's nice to have since something tangible to review. |
|  | Information Needs | In-person | Over the Internet is wonderful, but having it in person, where you say, “now, what about step one way or step two?” |
|  | Information Needs | Directive | [preference for training having] the device to walk through things. Having like the words on the device and say, “you need to do this.” |
|  | Training Needs | Mandates and Assessments | Before I could even like, put it in when they, when I signed up through the website, and like, made a profile and all that kind of thing, they had like school. There's like orientation modules that you have to complete, where you can like continue on to the other things. |
|  | Training Needs | Options | A training [about alerts] for sure, would be beneficial, and then, honestly, like a little cheat card [with all the alerts and their meaning], would be kind of nice if it was just like a little index card or something like that. |
|  | Information Needs | Alerts and Warnings | I like that idea like a wallet card almost like maybe having a little wallet card [with all safety alerts and warnings]. |
|  | Information Needs | Application and Applied Learning | I feel like give me all the information, but until I start using it and playing with it, then go back and have some questions, and maybe call the rep that showed me how to use it. or my doctor, or just getting online and doing the frequently asked questions or blogs. |
|  | Information Needs | Staged Approach | Basic starter, guide of “Here's how to do it” [use your device] for the first couple of days, and then something that's not too overwhelming. But then maybe something I could click through online on the website that would be videos or something interactive of how to go in depth on some of the more advanced features. |
|  | Information Needs | In-person | I personally like the [device] training with the person first, so I can have an opportunity ask questions, and then however else you want to give it to me for support. So, I would like, if someone was going to tell me about this app, I would like them to be fully knowledgeable about it, give me the training and then give me something in writing to follow-up that basically says the same thing or more than they just said, and then make themselves available to me to ask questions. |
|  | Information | Information Overload | There's too much information for me [with all the testing studies]. I don't really [need to know]... Try to keep things simple. |
|  | Information Needs | Staged Approach | Depends on where it is in the cycle. In the beginning, I like a lot of information to be able to hold my hand and walk through it. And then at that point, I just want to touch it and play with it and don't want to be like, “oh, hey, have you tried this?” I'll find that in a second. I'm just absorbing my information as it comes along. |
|  | Training Needs | Staged Approach | I just want to know at the beginning, explain it to me one time. I don't need to keep learning about it. But from the educator, whoever's giving me the device, I’d rather just talk about it, learn about it, move on. I don't ever read booklets or that kind of thing. I can kind of get lost in it. So if I didn't have a real life person, then just a little video that would maybe explain things [I need to know to safely use the device], like an optional video. |
|  | Information Needs | Site Navigation | I have an app that has a section that you can go to that's like, what's new, and you can go in there and you can navigate and see things. Like that diabetes is drinking from a fire hose, a ton of information all at once. And when you have new products, it's a lot of that. So having a little bit over time or your hands on experience. And then after a while, being able to go back and relearn. That's super helpful, because you'll remember more of it then. |
|  | Information Needs | Access and Availability | So having it accessible is really nice because then you can do a lot of it on your own. |
|  | Information Needs | Hands-on Application and Trial | There was a demo app I could use to do more of like the basic just exploring the menus. And then there was videos of how it worked. And then there was the online-basically like a class. It wasn't a video class, but I had to click through different slides with my mom and see how these things worked, and it gave me information. Then I had to answer some questions to make sure I was ready to use it |
|  | Information | Options | The answer is kind of yes to all of the types [videos, pamphlets, and different things that you could share with them], because you're gonna have different learners who use these. |
|  | Information | Access and Availability | If they're [the patients] up to accessing a website, a website with frequently asked questions, or a place to, you know, type in questions that they have, I think most folks would have some kind of access to that, but we take care of pretty poor folks, so I know how much it costs to keep my own Internet going. |
|  | Information Needs | Guidance | If the recommendations uh are not making sense to you, understand that you know you should contact your health, care provider and get more guidance. So yeah, just so you know, people get lost. Don't forget that they should ask for some outside help and not rely just on that app or that piece of technology. |
| **Troubleshooting and Maintenance Information** |  |  |  |
|  | Supports and Troubleshooting Resources | Preferences | I don't remember [troubleshooting information I received during initial training]. For the most part [I] feel like just having something I can look up myself, not having to go through calling 7 people and being on the phone [waiting]. Sometimes getting the right person is really frustrating. |
|  | Supports and Troubleshooting Resources | Access and Availability | If you have a 24-hour service, that's going to require some sort of a payment I would think. And with an app like that, especially for the older group who is Medicaid or whatever, really trying to manage costs and everything. So, would that have to come into play? I mean, would that deter people from doing something like this? |
|  | Supports and Troubleshooting Resources | Access and Availability | What would be kind of cool if it was possible, is, do like a zoom thing, if necessary, where you could, you know, meet with them and be able to like literally hold up your pump, or whatever. If it's having a problem, you'd be like, “This is what's happening. This is the alert that's coming. What does this mean?” |
|  | Supports and Troubleshooting Resources | Access and Availability | Maybe it is in some paperwork and I don't read the whole pamphlet every time, but I find it more helpful to have something pop up in the app itself, so that I have to look at it rather than just putting it in like some big long list of, “Here's some possibilities of what might happen.” |
|  | Supports and Troubleshooting Resources | Access and Availability | I personally like the [device] training with the person first, so I can have an opportunity ask questions, and then however else you want to give it to me for support. So, I would like, if someone was going to tell me about this app, I would like them to be fully knowledgeable about it, give me the training and then give me something in writing to follow-up that basically says the same thing or more that they just said, and then make themselves available to me to ask questions. |
|  | Supports and Troubleshooting Resources | Preferences and Options | So, you would like the step by step within the device, but you want the ability to turn it off once you have figured it out. |
|  | Supports and Troubleshooting Resources | Preferences and Options | We also need to have some videos that you know are Youtube videos [pop-up information videos] that also can hide [be in the background for when needed], there if you need it or you're confused. |
|  | Supports and Troubleshooting Resources | Preferences and Options | Basically, the same [in-person during the training and video alerts as back-up]. |
|  | Supports and Troubleshooting Resources | Access and Availability | [Maintenance and troubleshooting information should include] professional training, and we need a brochure. |
|  | Supports and Troubleshooting Resources | Preferences and Options | I'd want both a chat bot in the app, and maybe contact information, for example, a phone number or an email address to act as a backup [for information communication] |
|  | Supports and Troubleshooting Resources | Preferences and Options | It'd be nice if I had a separate app that could say, type in that error code and say, what do I do? If I can't find an online reference as to what I need to do or some kind of video as to how to fix it, then I'd better have an 800 number, or I can actually talk to a human being and say, "We got a problem, Houston." |
|  | Supports and Troubleshooting Resources | Preferences and Options | If I yeah again, within the algorithm, I would hope that if something's not going right, or the patient's perhaps doing something wrong, or they can't figure it out that they would have like a frequently asked questions. |
|  | Supports and Troubleshooting Resources | Access and Availability | That would be super helpful to you [the patient] if [information was] already there in the APP that could be built in. |
|  | Supports and Troubleshooting Resources | Access and Availability | I think the manufacturer should have a 24-hour seven day a week troubleshooting number. |
