# Supplementary material for: Artificial Intelligence in Health Care—Understanding Patient Information Needs and Designing Comprehensible Transparency: Qualitative Study
Source: JMIR AI. 2023 Jun 19;2:e46487. doi: 10.2196/46487 (PMC10851077; doi:10.2196/46487)
Supplement: Multimedia Appendix 3 [file ai_v2i1e46487_app3.docx]

**Multimedia Appendix 3.** **Safety concerns and needs**

|  | Themes | Sub-Themes | Representative Quotes |
| --- | --- | --- | --- |
|  |  |  |  |
| **Safety Needs** |  |  |  |
|  | Safety needs | Alerts | Maybe alerts that it is giving you this much insulin, because your blood sugar is high, information about why it's doing what it's doing, so you could learn from what it's doing. |
|  | Safety needs | Override | I would want to know how to shut it down. |
|  | Safety needs | Input controls | Yeah. I mean, this is going to run off of a base of history that you put in. And so, if you do put in a crazy number, it should say that's not possible or that's too many numbers. I feel like it should. After 90 days of putting information or 30 days of putting information in it should be able to realize that your top number of being high is going to be 300 and your lowest number for the type of person you are, might be 70. And if you put in a crazy number, like 500 and you meant to put 300, it could just say that doesn't make or something like that. |
|  | Safety needs |  | I would want to be darn sure that this thing [device] would never be able to [unexpectedly drop my blood sugar] do that. |
|  | Safety needs | Input controls | But I would want to know if I could do it [shut off the device] manually, if something went wrong, and I'd have to enter by hand if I could do that or not. |
|  | Safety needs | Input controls | Like a Max Bolus feature where it's like, “You can't give yourself more than this.” |
|  | Safety needs | Information sharing | I definitely think, having the capability to share [device information and readings] is good, because not everyone will want to share, but especially in the case of like a child and a parent, that that child Isn't, always going to know what to do, or especially depending on their age. |
|  | Safety needs | Information availability | One example is like it started to get inflamed where I had it on my arm, and then all of a sudden it said I was super low all the time, and it's like John here at 44 and I'm like, “That's bs I don't feel like that.” So then get some research and figured out that if there's information, then maybe your blood sugar locally can be because the cells are metabolizing a lot so that's true or not but it, it kind of gave me a reason to think. “Oh, maybe because my arms all red, I should change out my Libre, put it in a different place.” So yeah, kind of learned that that was a little bit more common for some people. |
|  | Safety features | Customization | To me it is really important what safety features are available, and what's the customization with those safety features. Good distinction sort of where we were headed with the next scenario, because, you essentially said that if it's giving you, and giving you just information or it's making a recommendation you sort of get annoyed about some of those safety features. |
|  | Safety Checks | Dashboard displays | No, I just do the test [the device] randomly to make sure everything is running right. But if I don't do it for three weeks, and there has been a problem for 3 weeks, then all my readings are messed up. So, is there a way for an alert to come right on the main screen that something's wrong? |
|  | Safety Needs | Information availability | Yeah, I think a hotline or somebody you can talk to if you are encountering any difficulties. |
|  | Safety Checks |  | So, I know with the pumps that are the closed hybrid group systems, most of them uh, because they talk to the sensor, have in those safety mechanisms whereby if there's a problem with the sugar's dropping, it's simply suspends the insulin dose. There's an in-built algorithm for safety. Or if someone's not doing something right, the sugars are high for hours and hours it goes into a safety more. Almost all the pumps have that these days. So yeah, I mean, I don't know if this needs to be that sophisticated. |
|  | Safety Needs | Control | Also, are they [patients with diabetes are] going to have the ability to override I think we always need the ability to override if things are low, just to stop. |
|  | Device Safety | Limitations | It's nice to know the [device safety] limitations that there are significant limitations. |
|  | Safety Checks |  | [safety need] something that would detect that the sensor was bad. |
|  | Safety needs | Training | formal safety training] might be helpful, but I just want to figure things out as I go. |
| **Safety Preferences** |  |  |  |
|  | Safety information | Training Preferences | I would like to receive safety information in person. |
|  | Safety Preferences | Alert Training | A training [about alerts] for sure, would be beneficial, and then, honestly, like a little check card [with all the alerts and their meaning], would be kind of nice if it was just like a little index card or something like that. |
|  | Safety Preferences | In-Person Training | I think it would be nice to have someone in person to just give me a little breakdown of, “Hey, these are really the most important things that you need to be aware of.” |
|  | Safety Training | New Product Orientation | I'd want [training about safety features] up front. That I'd want when I got the new product. |
|  | Safety Information | Preferences | [information required to safely use the device], probably in a booklet because I hate long startups. I hate to go through a new product and its take up to two hours to get started. |
|  | Safety Information | Access and Availability | If you're going to give me a written piece of paper, I want to make sure that it's something larger than a 0.5 font size. Because some of these things they put in boxes, they shrink it down to... We have to fit it in a box, and so everything goes down like a millimeter in height. I'm sorry, I just can't read a millimeter height. Not like I used to. Okay? I'm not a Marine or somebody that has great eyesight. I'm serious. You got to have something. That's why I prefer the online where I can at least... or the app, where I can go online and get all that stuff and read it. Because there I can change the font size and actually get something out of it rather than having to hire a 10-year-old kid next door to read it for me. |
|  | Safety Preferences | Alerts and Reporting | If the Dexcom is not reading correctly, I would hope it would just say the pump would start erroring and say hey you can't trust your Dexcom. |
|  | Safety Mandates | Requirements and Mandates | Pop up on the device itself. Otherwise also people don't otherwise won't do what you say [process updates and do required maintenance]. It seems like, um, yeah kind of built in. |
|  | Safety Training |  | If you're going to use this device like you're really going to need to know this [safety and basic device use information]. So, then you understand what's happening, and how to correctly use it |
