# Supplementary material for: Artificial Intelligence in Health Care—Understanding Patient Information Needs and Designing Comprehensible Transparency: Qualitative Study
Source: JMIR AI. 2023 Jun 19;2:e46487. doi: 10.2196/46487 (PMC10851077; doi:10.2196/46487)
Supplement: Multimedia Appendix 4 [file ai_v2i1e46487_app4.docx]

### Multimedia Appendix 4. Trust

|  | Themes | Sub-Themes | Representative Quotes |
| --- | --- | --- | --- |
|  |  |  |  |
| **Trust Accuracy** |  |  |  |
|  | Trust technology | Device selection | I trust my doctors enough [to select device for me]. |
|  | Trust technology | Device use | I trusted everything because of my doctor. It took me the first day [to trust the device] I just trusted. |
|  | Testing | Proven | I mean before a [device goes to] market or a product even hits the market. Typically, they do a whole bunch of testing. But it would be nice to know. You know this has been proven to work. This is like you can trust this. |
|  | Trust technology |  | At first it was hard for me to kind of trust it a little bit, because I was so used to doing the finger stick. And I just wasn't sure what the technology, what was going to happen? |
|  | Testing | Accuracy in Population | Did you just develop the app and never tested it on anybody and you're going to roll it out now and let people buy it and we don't know how accurate it is, or did you actually go through a process whereby you tested the product and it is comparable to others that may be in the category. |
|  | Testing and Use | Proven | I may be less likely to jump into new technology because I have the other technology I can compare it to. So, I'm using older information like we have our fingerstick glucometers that we can check and compare to our readings on a CGM or FGM. So, if there's a backup or something that you're familiar with to compare it to the new stuff, I think that makes it a lot easier to adapt. |
|  | Trust technology | Proven | I think that would depend for me if is this a machine learning? Does it learn the individual? If so, then yes, absolutely the diversity of that sample and how that machine learning algorithm actually works across groups is important. But if it's a more basic calculation that is kind of an already existing goal standard, then maybe less so, because if that would've happened on the back end in the development of that standard that exists. |
|  | Testing and Use | Reliable and Accurate | If there's a third-party device or app, I'm more interested in it. If there are ways you can change it or look at the information that it's going through or if it can be calibrated, if it's put out by the FDA or if it's something put out by a specific company or reputable company, I'd want to look at the research data. I'd want to see how many poor outcomes there were. I'd want to see what the reliability is and the accuracy of it, but the user information too. |
|  | Testing and Use | Reliable and Accurate | Because not everybody's diabetes is exactly the same. But it would be nice to see this person is getting really different results from me that are so different? They're not even proportional. What's going on with mine? Is there something broken? |
|  | Accuracy |  | I [provider] don't know that patients pay so much attention to its [the devices] accuracy. |
|  | Testing and Use |  | I want to make sure that that algorithm has been tested well in randomized control trials, to make sure that we're not getting. erroneous doses and stuff, especially when the AI technology taking over your dosing that's where the [glucose] level goes up to like get 10 but for something like in pen where it's really just a calculator taking all the data and doing the math for you, I don't need that tested I just need to know the math works. |
|  | Accuracy | Limitations | But if there are limitations to the accuracy, or if there are known things that would interfere with the readings, or the recommendations for some reason, so if there are limitations to the application. Suggest sharing that [information during training] |
|  | Accuracy | Tailored | I don't know that people using the devices necessarily think about that at all, and I think people who use the device care about them and how it is accurate or not accurate in them personally. |
| **Trust Endorsed By** |  |  |  |
|  | Endorsed | National organization | .. having something on the label that says the American Diabetes Association is a partner or approves it or you can talk to someone. |
|  | Endorsed | Peers | [In order to trust this device, I want to know] If there are, people [like me with my type of diabetes who] have actually used this device and [results]. |
|  | Endorsed | Medical Professional | I rely on my research but I, ultimately me, it really is going to be on the medical professional. |
|  | Endorsed | Medical Professional | I [rely on my healthcare provider]. |
|  | Endorsed | Medical Professional | I just trusted everything because of my doctors. |
|  | Endorsed | Medical Professional | [my source of information is my] Provider, other people with diabetes. |
|  | Endorsed | Pharmacists | Like you guys are doing it just. I just trust you guys [pharmacists]. |
|  | Endorsed | Manufacturer | I mean the manufacturer site if there's some great FAQs or whatever like that's the first place, I would go and look and then, just forums online of what other people have found. But yeah, usually I start with the manufacturer. |
|  | Endorsed | FDA | Then another thing that is important to me is if it's FDA approved, or not like if it's [the device] something that's vetted that way, then I trust it way more than I would trust something that doesn't carry that level of credibility and for me. |
|  | Endorsed | Peers | So, I prefer to have more personal reviews rather than the manufacturers like specifications. |
|  | Endorsed | Peers | I always want to know if someone else has used it, they've used it and what their opinion is of it. |
|  | Endorsed | Medical Professional | My number one [source of information] is my provider. |
|  | Endorsed | Peers | I feel like there needs to be a database for the past experiences of people that have used the app and how it worked for them. |
|  | Endorsed | National organization | I also think things such as American Diabetes Association. |
|  | Endorsed | National organization | Like diabetes group sources, like VA or the CDC educator groups where I got my information. |
